# Supplementary material for: A Cap-Optimized mRNA Encoding Multiepitope Antigen ESAT6 Induces Robust Cellular and Humoral Immune Responses Against Mycobacterium tuberculosis
Source: Vaccines (Basel). 2024 Nov 9;12(11):1267. doi: 10.3390/vaccines12111267 (PMC11599153; doi:10.3390/vaccines12111267)
Supplement: Supplementary file 1 [file vaccines-12-01267-s001.zip › Figure S1.pdf]

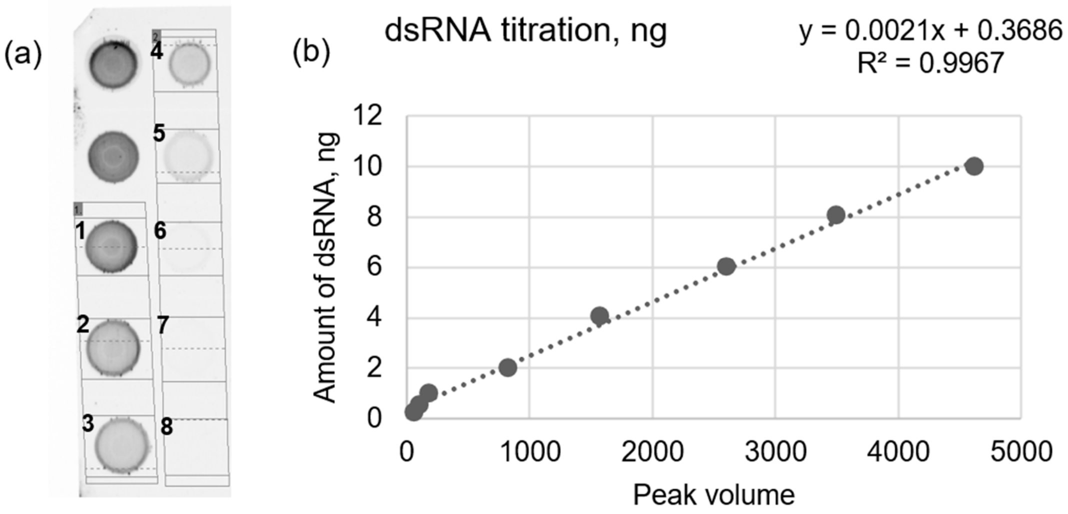

Figure S1. Preparation of a series of dsRNA standards for estimating the amount of dsRNA in mEpitope-ESAT6 mRNA sample. (a) Samples of prepared dsRNA in the amount were applied to nitrocellulose membrane. (b) Calibration curve plotted against signal intensity values (Peak Volume). GelAnalyser 23.1.1 application was used to analyze the dot-blot results.
