# Supplementary material for: A Cap-Optimized mRNA Encoding Multiepitope Antigen ESAT6 Induces Robust Cellular and Humoral Immune Responses Against Mycobacterium tuberculosis
Source: Vaccines (Basel). 2024 Nov 9;12(11):1267. doi: 10.3390/vaccines12111267 (PMC11599153; doi:10.3390/vaccines12111267)
Supplement: Supplementary file 1 [file vaccines-12-01267-s001.zip › Figure S2.pdf]

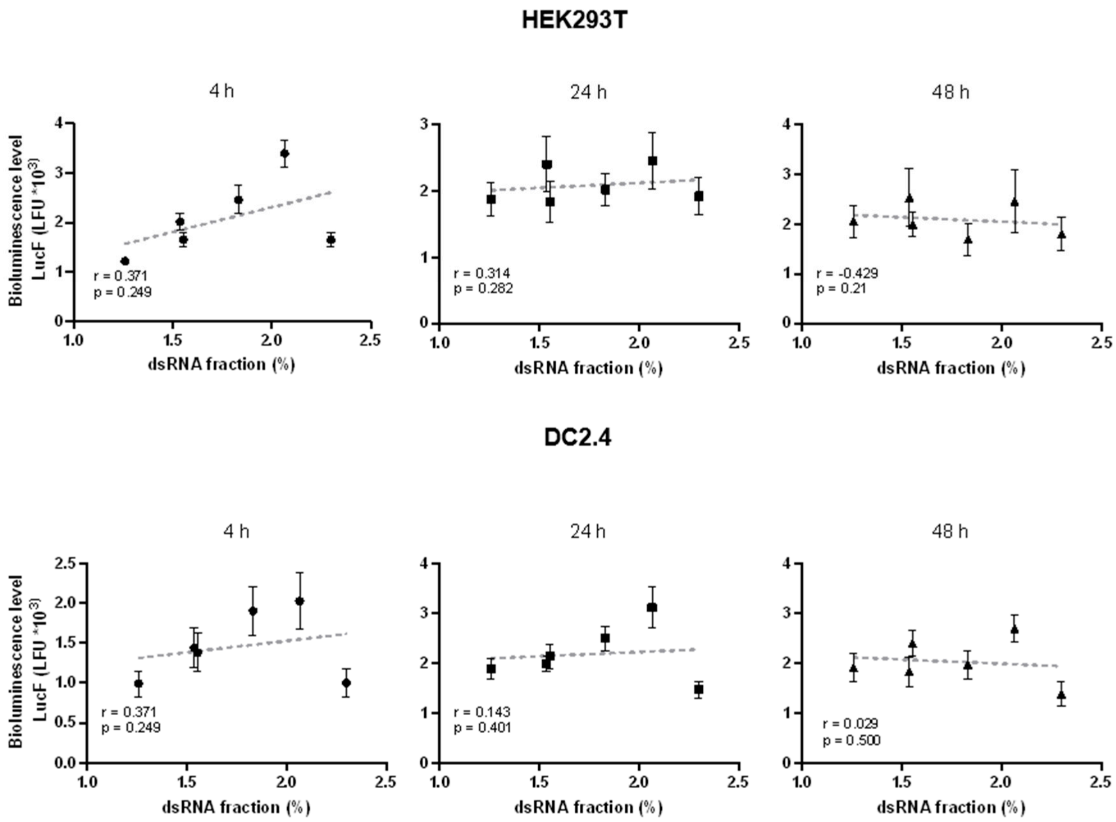

Figure S2. Analysis of correlations between the amount of dsRNA and normalized luciferase bioluminescence intensity
