# Supplementary material for: A Cap-Optimized mRNA Encoding Multiepitope Antigen ESAT6 Induces Robust Cellular and Humoral Immune Responses Against Mycobacterium tuberculosis
Source: Vaccines (Basel). 2024 Nov 9;12(11):1267. doi: 10.3390/vaccines12111267 (PMC11599153; doi:10.3390/vaccines12111267)
Supplement: Supplementary file 1 [file vaccines-12-01267-s001.zip › Sequence S1.pdf]

Sequence S1

Nucleotide sequence of mEpitope-ESAT6 mRNA

$m_2^{7,3\text{-}O}$GpppGmGGAAAUACUCUCUUCGCAUCGCUGUCUGCGAGGGCCAGCUGUU
GGGCUCGCGGUUGAGGACAAACUCUUCGCGGUCUUUCCAGUACUCUUGGAUCG
GAAACCCGUCGGCCUCCGAACGGUACUCCGCCACCGAGGGACCUGAGCGAGUC
CGCAUCGACCGGAUCGGAAAACCUCUCGAGAAAGGCGUCUAACCAGUCACAGUC
GCAAGAAGCUUAGCAUUCCGGUAACUGUUGGUAAAGCCACCAUGUGGAACUUCG
CCGGCAUAGAGGCCGCCGCCAGCGCCAUACAGGGCAAGAAGCAGAGCCUG
ACCAAGCUGGCCGCCGCCUGGGGCGGGAGCGGCAAGAAGCUGAACAACGCCCU
GCAGAACCUGGCCCGCACCAUAAGCGAGGCCGCCGCCUACCUGCUCGACGAGG
GCAAGCAGAGCCUGGCCGCCUACGCCGCCUGGGGCGGGAGCGGCAGCGAGGC
CUACGCCGCCUACAUAGUGGGCAUAGUGGCCGGCCUGGCCGUGCUGAUAGUGG
UCGUGAUAGGCGCCGUGGUCGCCACCGUGAUGUGCCGCAGGAAGAGCUCCGGC
GGGAAGGGCGGGAGCUACAGCCAGGCCGCCAGCUCCGACAGCGCCCAGGGCAG
CGACGUGAGCCUGACCGCCUGAUAAGCUGGAGCCUCGGUGGCCUAGCUUCUUG
CCCCUUGGGCCUCCCCCCAGCCCCUCCUCCCCUUCCUGCACCCGUACCCCCGU
GUCUUUGAAUAAAGUCUGAGUGGGCGGCAGAUCUGACUGAAAAAAAAAAAAAAA
AAAAAAAAAAAAAAAAAAAAAAAAAAAAAAAAAAAAAAAAAAAAAAAAAAAAAAAAAA
AAAAAAAAAAAAAAAAAAAAAAAAAAAAAAAAAAAAAAAAA

The synthetic analog of 5'-cap (cap-1) is highlighted in purple.

The 5' untranslated sequence is highlighted in red

The coding sequence is highlighted in black (decoded below)

The 3' untranslated sequence is highlighted in blue

The polyA tail sequence (114A) is highlighted in green

Amino acid sequences of the protein product encoded by mRNA

MWNFAGIEAAASAIQGKKKQSLUKLAAAWGGSGKKLNNALQNLARUISEAAAYLLDEG
KQSLAAYAAWGGSGSEAYAAY**IVGIVAGLAVLIVVVIGAVVAUVMCRRKSSGGKGGS
YSQAASSDSAQGSDVSLUA***

A five epitopes are encoded in total. Linker sequences (KK or AAY) connecting different epitopes are highlighted in red. The signal peptide, a sequence of the MHC class I transport signal, which ensures co-localization of the target protein with histocompatibility complexes in various endocytic compartments (Golgi apparatus, endoplasmic reticulum, endosomes) and on the cell membrane surface, significantly enhancing antigen presentation, is shown in bold.
