# Supplementary material for: A Cap-Optimized mRNA Encoding Multiepitope Antigen ESAT6 Induces Robust Cellular and Humoral Immune Responses Against Mycobacterium tuberculosis
Source: Vaccines (Basel). 2024 Nov 9;12(11):1267. doi: 10.3390/vaccines12111267 (PMC11599153; doi:10.3390/vaccines12111267)
Supplement: Supplementary file 1 [file vaccines-12-01267-s001.zip › Table S2.pdf]

Table S2. Components of the transcriptional mix for CapAG capped mRNA synthesis (total volume 50 mkl)

	Non-capped mRNA		CapAG-capped mRNA					
Cap:GTP ratio ->	-		0,4:1		0,8:1		1:1	
Components	Volume, µl	Final concentration	Volume, µl	Final concentration	Volume, µl	Final concentration	Volume, µl	Final concentration
Buffer (5x)	10	x1	10	x1	10	x1	10	x1
DTT (10x)	5	x1	5	x1	5	x1	5	x1
GTP, 30 мМ	5	3 мМ	5	3 мМ	5	3 мМ	5	3 мМ
UTP, 30 мМ	5	3 мМ	5	3 мМ	5	3 мМ	5	3 мМ
CTP, 30 мМ	5	3 мМ	5	3 мМ	5	3 мМ	5	3 мМ
ATP, 30 мМ	5	3 мМ	5	3 мМ	5	3 мМ	5	3 мМ
CapAG 100 мМ	-	-	0,6	1.2 мМ	1.2	2.4 мМ	1.5	3 мМ
Water sterile	to 50 µl	-	to 50 µl	-	to 50 µl	-	to 50 µl	-
DNA template	X	20 ng/ µl	X	20 ng/µl	X	20 ng/µl	X	20 ng/µl
Ribo-Care, 40 U/µl	1.25	1 U/ µl	1.25	1 U/µl	1.25	1 U/µl	1.25	1 U/µl
T7-polimerase, 300 U/mkl	3	18 U/µl	3	18 U/µl	3	18 U/µl	3	18 U/µl
Pyrophosphotase, 100 U/ml	1	0.002 U/µl	1	0.002 U/µl	1	0.002 U/µl	1	0.002 U/µl
