# Supplementary material for: A Cap-Optimized mRNA Encoding Multiepitope Antigen ESAT6 Induces Robust Cellular and Humoral Immune Responses Against Mycobacterium tuberculosis
Source: Vaccines (Basel). 2024 Nov 9;12(11):1267. doi: 10.3390/vaccines12111267 (PMC11599153; doi:10.3390/vaccines12111267)
Supplement: Supplementary file 1 [file vaccines-12-01267-s001.zip › Table S3.pdf]

Table S3. Summary of translation efficiency and dsRNA content for reporter RNAs carrying with cap-analogs at different cap to GTP ratios.

Cap-analog	Cap:GTP ratio	dsRNA fraction (%)	Total protein expression	
			HEK293T	DC2.4
$m_2^{7,3\text{-}O}$GpppG	2:1	1.75 ± 0.4	0.13 ± 0.10	0.10 ± 0.07
$m_2^{7,3\text{-}O}$GpppG	4:1	1.69 ± 0.4	1.00 ± 0.58	1.00 ± 0.40
$m_2^{7,3\text{-}O}$GpppG	8:1	1.45 ± 0.1	0.18 ± 0.11	0.20 ± 0.12
$m_2^{7,3\text{-}O}$GpppGmG	2:1	1.57 ± 0.3	2.05 ± 1.02	2.11 ± 0.97
$m_2^{7,3\text{-}O}$GpppGmG	4:1	2.02 ± 0.1	2.77 ± 1.61	2.62 ± 1.26
$m_2^{7,3\text{-}O}$GpppGmG	8:1	1.50 ± 0.1	2.31 ± 1.45	1.73 ± 0.84
$m_2^{7,3\text{-}O}$GpppAmG	0.4:1	1.66 ± 0.6	1.79 ± 0.90	1.26 ± 0.69
$m_2^{7,3\text{-}O}$GpppAmG	0.8:1	1.53 ± 0.3	1.71 ± 0.88	1.58 ± 0.88
$m_2^{7,3\text{-}O}$GpppAmG	1:1	1.24 ± 0.3	1.83 ± 0.83	1.97 ± 0.94

Total protein expression of luciferase mRNA with these 5'-capped analogues, normalized to mRNA expression of ARCA-capped.
